# Extracellular Vesicles Derived from Breast Cancer Cells: Emerging Biomarkers of Tumor Progression and Metastasis

**DOI:** 10.3390/biom15081195

**Published:** 2025-08-19

**Authors:** Sona Bernatova, Andreas Nicodemou, Michaela Cehakova, Lubos Danisovic, Martin Bohac

**Affiliations:** 1 Institute of Medical Biology, Genetics and Clinical Genetics, Faculty of Medicine, Comenius University, Sasinkova 4, 811 08 Bratislava, Slovakia; andreas.nicodemou@fmed.uniba.sk (A.N.); michaela.cehakova@fmed.uniba.sk (M.C.); lubos.danisovic@fmed.uniba.sk (L.D.); martin.bohac@fmed.uniba.sk (M.B.); 2 2nd Department of Oncology, Faculty of Medicine, Comenius University, Klenova 1, 833 10 Bratislava, Slovakia

**Keywords:** breast cancer, extracellular vesicles, cancer biology

## Abstract

Breast cancer (BC) remains one of the most prevalent and life-threatening malignancies worldwide, marked by significant heterogeneity and complex mechanisms of progression. Despite major advances in understanding its molecular and cellular basis, the processes driving tumor progression and metastasis continue to challenge effective treatment. Among the emerging research areas, extracellular vesicles (EVs) have gained considerable attention for their key role in intercellular communication and their contribution to cancer biology. In BC, tumor cell-derived EVs are implicated in multiple processes that promote disease progression, including tumor growth, remodeling of the tumor microenvironment, and facilitation of metastasis. By transferring oncogenic signals to recipient cells, EVs critically shape the metastatic niche and support the spread of cancer cells to distant organs. Recent studies highlight the diverse functions of BC-derived EVs in modulating immune responses, inducing angiogenesis, and enhancing cancer cell invasiveness. This review explores the role of BC-derived EVs in tumor progression and metastasis. We discuss their molecular composition, mechanisms of action, and impact on the tumor microenvironment, aiming to provide insights into their role in BC pathophysiology and discuss potential clinical applications. A deeper understanding of the complex interplay between EVs and cancer progression may pave the way for innovative strategies to combat BC and improve patient outcomes.

## 1. Introduction

In recent years, EVs have emerged as promising biomarkers for the early detection, prognosis, and monitoring of BC, owing to their unique molecular cargo and stability in bodily fluids [1,2,3]. Significant progress has been made in identifying and validating EV-derived biomarkers for BC. These advances are reshaping diagnostic paradigms by offering non-invasive, highly sensitive tools that complement—or even surpass—traditional biopsy-based approaches [4,5,6].

The ability to isolate and analyze EVs from easily accessible fluids enables non-invasive “liquid biopsies,” which are particularly valuable for early cancer detection and longitudinal disease monitoring. This approach not only reduces patient discomfort but also provides a more comprehensive representation of tumor heterogeneity, in contrast to tissue biopsies, which typically sample only a small portion of the tumor region [7].

Integrating EV-based biomarkers into personalized treatment strategies facilitates more precise and adaptive cancer management. As EV isolation and analytical technologies continue to evolve, and as additional biomarkers are validated in large-scale clinical trials, EV-based diagnostics are poised to become a cornerstone of precision oncology, offering renewed hope for improved patient outcomes.

Recent studies have identified a wide array of EV-derived biomarkers, including proteins, nucleic acids (e.g., DNA, microRNA (miRNA), messenger RNA (mRNA), circular RNA (circRNA), long non-coding RNA (lncRNA), small non-coding RNA (sncRNA), RNAs, transfer RNA-derived fragments (tRFs)), and metabolites. These molecules provide crucial insights into tumor biology, enabling early detection, subtype classification, and real-time monitoring of treatment response and disease progression [2,8,9,10].

The primary objective of this article is to comprehensively investigate the role of EVs derived from BC cells in tumor progression and metastasis, with a particular focus on their molecular cargo and their contribution to subtype-specific pathophysiology. The review also aims to explore the diagnostic, prognostic, and therapeutic potential of EVs in precision oncology.

## 2. Breast Cancer

BC is currently the most diagnosed cancer among women worldwide. Even when considering both sexes, it remains the second most prevalent cancer and the fourth leading cause of cancer-related death globally, as of 2022 [11].

Approximately one in eight to ten women will be diagnosed with BC during their lifetime [12], whereas less than 1% of men are diagnosed with the disease [13]. In total, there were 2.3 million new cases and 670,000 deaths due to female BC globally in 2022. Annual incidence rates are increasing by 1–5%.

A comparison between countries with a high and low Human Development Index (HDI and LDI) reveals that HDI countries show declining mortality rates. At the same time, LDI countries continue to experience an increase in BC-related deaths [14].

From a national perspective, in Slovakia, BC is the leading cause of cancer-related death among women, accounting for 27% of all female cancer cases. Regional disparities exist, with the capital city, Bratislava, reporting higher incidence rates compared to other regions. According to the European Cancer Information System, a 25% increase in total cancer cases is projected between 2022 and 2040, although this estimate is not specific to BC [15].

These data underline the ongoing global threat posed by BC and the pressing need for improvements in prevention, diagnostics, and treatment. As of now, PubMed lists 548,965 articles related to BC, with the earliest dating back to 1789—and the number continues to grow.

BC is not a singular disease with a single cause or outcome. It is a heterogeneous and multitype condition involving numerous initiating events and variations in biological and clinical behavior. This complexity is rooted in diverse genetic alterations that disrupt the function and regulation of specific genes and cellular processes [16,17,18].

Numerous factors contribute to BC oncogenesis, including genetic predisposition, environmental exposures, and lifestyle influences [19,20]. Interestingly, elements such as circadian rhythm disruptions, tumor-associated microbiota, and cancer stemness have also been identified as drivers of disease progression, adding further layers to the already intricate pathophysiology of BC [21].

According to the intrinsic classification proposed by Perou and Sørlie in 2000 [22], (BC) can be divided into four molecular subtypes: Luminal A and Luminal B (both characterized by estrogen receptor (ER) expression), basal-like (lacking ER, progesterone receptor (PR), and epidermal growth factor receptor 2 (HER2) expression—commonly referred to as triple-negative breast cancer (TNBC)), and HER2-enriched (lacking ER but overexpressing HER2).

The most common histological types of BC based on tissue origin are ductal carcinoma and lobular carcinoma, both of which are invasive lesions. Their non-invasive counterparts include ductal carcinoma in situ and lobular carcinoma in situ.

In current clinical practice, BC is typically classified into five subtypes based on histological and molecular characteristics, including the expression of ER, PR, HER2, and the proliferation marker Ki67. Tumors expressing ER and/or PR are classified as *hormone receptor-positive*, while those lacking ER, PR, and HER2 are categorized as *triple-negative* [23].

Based on the PAM50 gene expression classifier, which builds on these molecular features, five subtypes are identified: Luminal A, Luminal B, HER2-enriched, basal-like, and normal-like [24,25,26,27].

Currently, the World Health Organization (WHO) recognizes approximately 20 distinct histological types of breast carcinoma, in addition to invasive ductal carcinoma, which remains the most common subtype [28].

**Figure 1:**
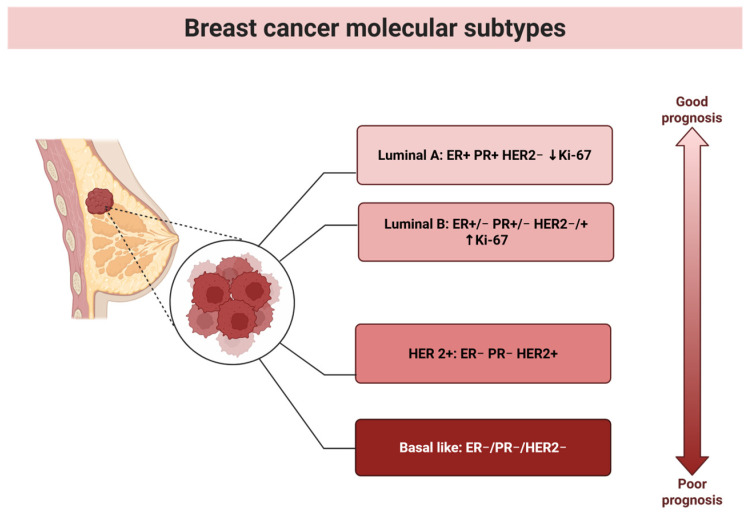
BC molecular subtypes and disease aggressiveness. BC is divided according to the presence or absence of specific receptor expression (Figure 1). Based on disease prognosis, we can rank the molecular subtypes from those with the best to those with the worst prognosis. Luminal A BC represents a molecular subtype characterized by the expression of ER and PR, the absence of HER2 receptor expression, low expression of Ki-67, and a relatively favorable disease prognosis. The second, more aggressive molecular subtype is Luminal B, characterized by the presence of ER and/or PR, and can be either HER2-positive or HER2-negative. It is also characterized by high Ki-67 expression. The third subtype is HER2-positive BC, characterized by the lack of progesterone and estrogen receptor expression and the presence of HER2 receptor expression. The molecular subtype with the worst disease prognosis is TNBC, which is characterized by the absence of estrogen, progesterone, and HER2 receptor expression.

Despite these classification systems, further refinement is needed to better distinguish between the diverse forms of BC (Figure 1), thereby enabling more accurate diagnosis, prognosis, and personalized treatment strategies.

## 3. Extracellular Vesicles

**Figure 2:**
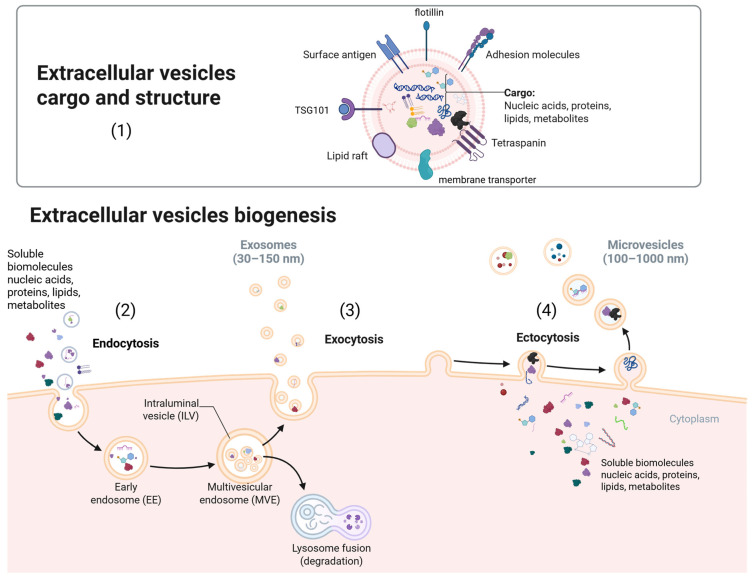
Extracellular vesicle cargo, structure, and biogenesis. (**1**) EVs are particles formed from a lipid bilayer, characterized by various membrane-associated regulatory biomolecules and specific internalized cargo consisting of regulatory biomolecules. In the cell, EVs undergo a process of biogenesis, which begins with endocytosis (**2**), where various soluble biomolecules and EVs are internalized by the cell into the cytoplasm, leading to the formation of early endosomes. These then progress to form multivesicular endosomes (MVEs) consisting of intraluminal vesicles, where the packaging with various molecules occurs. The subsequent pathway continues either by the MVE content being tagged and fused with lysosomes, where degradation of unnecessary biomolecules takes place, or by the MVE content being secreted in the form of exosomes containing regulatory biomolecules through the process of exocytosis (**3**) into the extracellular space. Another method of EV genesis is ectocytosis (**4**), also known as membrane shedding, during which soluble bioactive molecules are packaged into the cell membrane and subsequently released into the extracellular space in the form of microvesicles or ectosomes.

EVs are a heterogeneous group of membrane-bound particles that include several subtypes based on their size and origin (Figure 2). These include apoptotic bodies (500 nm to 2 µm in diameter), microvesicles (100 nm to 1 µm), exosomes and small extracellular vesicles (sEVs) (30 to 150 nm) [29,30], and oncosomes (1 to 10 µm) [31,32]. However, current literature states different size limits for each category. In this regard, the Minimal Information for Studies of Extracellular Vesicles (MISEV) guidelines should be taken into consideration. An updated version was released in December 2023, which states the following EV size categories: sEVs under 200 nm in diameter, and large EVs over 200 nm in diameter. Other terms, such as exosomes, ectosomes or microvesicles are not recommended unless the subcellular origin is demonstrable [33].

In an alternative classification system, EVs are broadly divided into three main categories, with oncosomes considered a subtype of larger vesicles [34]. Other recognized subtypes in this framework include large dense core vesicles, membrane blebs, outer membrane vesicles, and prostasomes [35,36,37]. Additional specialized forms such as tolerosomes, proteasomes, prominosomes, migrasomes, exomeres, exophers, and mitovesicles have also been described [38,39].

EVs are found in prokaryotes, eukaryotes, and plants [36]. These vesicles formed by a lipid bilayer, are incapable of replication and do not contain a functional nucleus [37]. EVs are secreted into the extracellular space [30]. Microvesicles, also known as ectosomes [40], are formed directly from the plasma membrane and contain cargo from the cytoplasm [31,41]. Exosomes are formed by the fusion of multivesicular bodies (MVBs) with the plasma membrane, where MVB produces smaller vesicles (exosomes). Dying cells produce apoptotic bodies, which are present in higher quantities in the body compared to exosomes or microvesicles. Under specific conditions, their composition can vary across different biological fluids [31,42,43,44]. Oncosomes are large EVs formed from membrane protrusions and are primarily secreted by malignant cells [31,32,42]. Another type of EVs, argosomes, differs from exosomes in function. They carry morphogens—proteins that form concentration gradients within tissues and contribute to signal transduction processes, ensuring correct cell positioning during the development of multicellular organisms. Argosomes are believed to facilitate cargo transfer from donor to recipient cells [45,46]. Apoptotic blebs are membrane protrusions that arise when the plasma membrane detaches from the cortical cytoskeleton, usually due to increased hydrostatic pressure during cell contraction. These blebs, formed during programmed cell death, cover the entire cell surface and enclose chromatin and organelles. As apoptosis progresses, these blebs fragment into membrane-enclosed apoptotic bodies [46,47]. Tolerosomes are produced by intestinal endothelial cells [48], proteasomes are multistructural complexes designed for protein degradation [49,50], and prominosomes are associated with the plasma membrane, containing the prominin protein, and are considered organizational elements of the plasma membrane [51,52]. EVs are classified based on biogenesis, secretion pathways, size, content, and function [31,36,46]. Currently, markers specifying whether an EV originates from an endosome (exosomes) or the plasma membrane (ectosomes—microparticles/microvesicles) have not been precisely described. Assigning EVs to a specific subtype based on their biogenesis mechanism is complicated without real-time imaging [37,53,54]. Therefore, if EV identification based on precise markers of cellular origin is not possible, EVs should be identified according to the following categories: physical characteristics such as size: sEVs < 100 nm or <200 nm, and large or medium EVs > 200 nm; density—low, medium, or high, ranging from 1.06 to 1.21 g/mL [37,54], or 1.13 to 1.19 g/mL in sucrose [46,55]; biochemical content such as CD63^+^/CD81^+^-EVs, or EVs labeled with annexin V; or based on the description of the cell’s state and origin—EVs from podocytes, hypoxic EVs, large oncosomes, or apoptotic bodies [37]. As previously thought, EVs are a waste management system [56]; they have been identified as a communication interface delivering signaling biomolecules such as lipids, proteins, nucleic acids [57,58], thus driving key metabolic processes within functional physiology [59,60], creating a connection between EVs and functional pathways of various cancer physiologies including tumor progression and metastasis [61,62,63].

## 4. Extracellular Vesicles and Cancer

EVs are recognized as key mediators of intercellular communication, significantly influencing metabolic pathways in living organisms. As integral components of signaling networks, they are associated with a wide range of regulatory processes in biological systems. EVs play a critical role in cancer initiation, progression, and even regression of pathological states [64,65]. Their importance lies in their ability to transfer genetic and epigenetic regulatory factors between cells [66,67]. In addition to nucleic acids, EVs carry other bioactive molecules, such as proteins, which can be either enclosed within the vesicle or associated with its membrane [68,69]. Notably, lipids represent another major class of regulatory cargo, playing essential roles in cellular metabolism and the intricate mechanisms of EV-mediated communication [70]. These biomolecules, whether encapsulated in the EVs or present on their surface, are being intensively studied for their involvement in oncopathological processes, many of which are already known to contribute to cancer development.

In the following section, we highlight specific examples of EV cargo—comprising nucleic acids, proteins, and lipids—that contribute directly or indirectly to oncopathological processes (Table 1).

### 4.1. RNA EV Cargo Regulating Cancer Progression and Metastasis

According to Lei et al. [71], the lncRNA H19 is secreted into EVs, promoting resistance to epidermal growth factor receptor (EGFR) tyrosine kinase inhibitors in non-small-cell lung cancer cells. Additional evidence suggests that the presence of AGAP2 antisense RNA1 in EVs may contribute to resistance against human HER2 inhibitors [72]. Wang et al. [73] reported that in patients with colorectal cancer (CRC), EVs derived from the liver were enriched with miRNAs that regulate YAP signaling. These miRNAs suppressed LATS2 expression, thereby promoting CRC liver metastasis.

In renal cell carcinoma (RCC), EVs carrying circRNA circEHD2—found to be upregulated in tumor tissues—contribute to metastasis by inducing the secretion of the proinflammatory cytokine IL-6 [74]. Given that inflammation is a well-established contributor to cancer development [75,76,77], these findings further highlight the role of EVs in tumor-promoting inflammation. Another study focused on cancer-associated fibroblasts (CAFs), which are known to facilitate tumor growth and progression. A specific lncRNA was identified in EVs that correlates with CAF enrichment in bladder cancer [78]. Specifically, miR-181a-5p, present in EVs derived from CRC cells, has been shown to promote liver metastasis by activating hepatic stellate cells and modifying the tumor microenvironment [79]. In contrast, according to Luo et al. [80], miR-29a-3p-enriched EVs secreted by myocytes enhance anti-tumor immunity by downregulating collagen composition in tumors. Notably, the secretion of EVs containing miR-29a-3p is strongly upregulated by physical exercise.

There is also evidence for the presence of EV-associated cell-free mRNA (cf-mRNA) in various cancer types. Specifically, mRNAs 136, 199, 462, 151, and 105 were found to be significantly enriched in cancer samples compared to healthy controls [81]. Furthermore, miR-891b, which targets the phosphoserine aminotransferase 1 (PSAT1) gene, has been implicated in several cancers by regulating PSAT1 expression, potentially contributing to metastasis through EV-mediated signaling [82].

To this point, four types of RNA—miRNA, lncRNA, circRNA, and cf-mRNA—have been highlighted for their roles in EV cargo linked to oncopathology. However, additional RNA species are also present in EVs and are relevant to cancer biology, including mRNA [83], transfer RNA (tRNA) [84,85], small nuclear RNA (snRNA) [86], small nucleolar RNA (snoRNA) [87], mitochondrial RNA (mtRNA) [88,89], piwi-interacting RNA [90,91], Y RNAs [92], and retrotransposon-derived RNAs [93].

As these examples illustrate, RNA cargo in EVs is highly diverse and contributes through multiple mechanisms to tumor initiation, progression, and metastasis. Similar complexity and oncogenic potential have been observed in EV-associated proteins, DNA, lipids, and metabolites, further underscoring the multifaceted role of EVs in cancer biology.

### 4.2. DNA EV Cargo and Its Presence in Cancer

DNA cargo in EVs has emerged as a valuable tool in cancer diagnostics and prognostics. The phospholipid bilayer of EVs protects encapsulated nucleic acids from degradation by nucleases present in biological fluids. This protection makes EV-associated DNA a more stable and reliable candidate for cancer monitoring compared to freely circulating DNA (cfDNA) [94], enabling more precise and sensitive mutation detection [95,96,97,98,99,100]. DNA derived from EVs includes large-sized genomic fragments and tumor-specific mutant DNA [95]. In a study by Clancy and coworkers, a significant elevation of double-stranded DNA (dsDNA) was observed in EVs isolated from metastatic melanoma cell lines, compared to the primary A375P line and its metastatic variant A375-MA2 [99]. Another study described the role of oncogenic H-Ras—a GTPase involved in cell cycle regulation—in driving vesicle formation in intestinal epithelial cells. This process results in the release of sEVs containing chromatin-associated dsDNA, representing the host genome modified by oncogenic mutations. These EVs, which include DNA carrying the H-ras mutation, may interact with and influence normal cells [100]. Balaj et al. demonstrated that EVs released from tumors carry DNA reflecting tumor-specific features, such as amplification of the c-Myc oncogene [93]. Similarly, Kahlert et al. [101] identified dsDNA in serum-derived EVs from pancreatic cancer patients. These vesicles contained fragments over 10 kb in length, allowing for the detection of KRAS and TP53 mutations, thereby supporting the application of serum-based liquid biopsy for pancreatic cancer diagnosis.

Guescini and coworkers were the first to report the presence of mitochondrial DNA (mtDNA) in EVs secreted by glioblastoma cells and astrocytes. Their study showed that approximately 5% of total cellular mtDNA could be found in EVs, supporting the concept of horizontal mtDNA transfer between cells [102]. Overall, the field of EVs with DNA cargo is rapidly evolving, with a growing body of literature highlighting its complexity and diagnostic potential.

### 4.3. Protein EV Cargo in Cancer

Proteins involved in the regulation of oncopathology are found either within the intraluminal space of EVs or associated with their membrane surfaces [62]. According to Urabe et al., the EV-associated transmembrane protein CUB domain-containing protein 1 (CDCP1) acts as an inducer of osteoclastogenesis, thereby promoting metastasis in patients with prostate cancer [103]. A strong link between aging and melanoma progression was highlighted in the study by Hüser et al. [104]. The authors demonstrated that aging is associated with reduced expression of CD9—a key regulator of EV cargo sorting—which was diminished both in EVs and parental cells. This reduction was accompanied by an increase in angiopoietin-like protein 2, a potent pro-angiogenic factor, thereby facilitating angiogenesis and tumor progression. Further insights into the oncogenic potential of EVs were provided by McAtee et al. [105]. Their study revealed that EVs can induce the formation of filopodia, cellular structures essential for polarization, adhesion, and migration. The EVs responsible for this effect were enriched in the TGF-β family co-receptor endoglin, which modulates filopodia dynamics. Additionally, the presence of the transmembrane protein thrombospondin type 1 domain-containing 7A in EVs was shown to further enhance filopodia formation. In a pivotal study by Peinado et al. [106], several EV-associated proteins were proposed as potential biomarkers for melanoma progression. TYRP2, VLA-4, and HSP70 were significantly elevated in EVs from stage IV melanoma patients, while only TYRP2 showed significant elevation in stage III, correlating with metastatic potential. The study also demonstrated that the mesenchymal–epithelial transition (MET) oncoprotein, when transferred via EVs to bone marrow progenitor cells, could induce genetic and epigenetic changes associated with metastatic spread. Mass spectrometry analyses by Mears et al. [107] and Staubach et al. [108] confirmed that EVs secreted by melanoma and human breast carcinoma cells also carry mtDNA cargo, further supporting their role in horizontal transfer of oncogenic material. Overall, a growing body of literature underscores the presence and functional relevance of diverse protein molecules in EVs that are directly involved in cancer regulation and progression.

### 4.4. Lipid EV Cargo in Cancer

Lipids are one of the main components of EVs. EVs are rich in cholesterol (CHL), diglycerides, glycerophospholipids, sphingolipids and glycosylceramides [109,110]. In addition, they also contain bioactive lipids, such as phospholipase C and A2 [111,112,113]. These bioactive lipids, when transported by exosomes, can exert regulatory functions in recipient cells [114,115]. In a study by Skotland et al. [116], a specific combination and ratio of phosphatidylserine (PS) to ceramide (CER), as well as alterations in the levels of phosphatidylcholine (PC) and CHL within exosomes, were shown to reflect metabolic dysregulation in prostate cancer cells. These findings support the use of sEV-derived lipids from patient samples as potential biomarkers for prostate cancer diagnosis and therapy.

A separate study focusing on the metastatic prostate cancer cell line PC-3 quantified over 300 distinct lipids present in sEVs secreted by these cells. Glycosylated phospholipids were identified as promising biomarker candidates for malignant prostate pathology. Furthermore, the lipidome of sEVs differed significantly from that of the parent cells, with sEVs showing higher concentrations of glycosphingolipids, sphingomyelin SM, CHL, and PS [117]. Another study compared EV samples from patients with lung cancer, benign conditions, and healthy individuals. In EVs from lung cancer patients, elevated levels of lipids such as Cer(42:1), triglyceride(54:2), PC(24:0), PC-O(32:0), and lysophosphatidylcholine (LysoPC)(20:1) were detected. Increased levels of cholesteryl ester Cer(19:2) were found in patients with both benign and malignant conditions, compared to healthy controls. Conversely, higher levels of Cer(38:6), PC(42:2), PC(36:5), and PC(38:7) were observed in EVs from healthy individuals. The study also showed that lipid composition differs between serum and EV isolates, further emphasizing the functional specificity of EV cargo. However, due to methodological inconsistencies in EV isolation and normalization, as well as sample variability, it was not possible to construct a statistically robust multiplex lipid biomarker panel. Nonetheless, such a panel holds potential for future diagnostic or prognostic use [118]. In RCC, Del Boccio et al. [119] identified 48 EV-derived lipids with statistically significant alterations. Of these, 22 lipids were elevated and 26 were decreased under pathological conditions compared to healthy samples, suggesting their potential utility as biomarkers. Overall, growing evidence links lipid cargo variations in EVs with different types of cancer. These findings support the development of EV-based diagnostics, prognostics, and therapeutic strategies in oncology.

### 4.5. Metabolite EV Cargo in Cancer

In the study by Palviainen et al. [120], the authors provided the first evidence that metabolites carried by EVs reflect the altered metabolic state of various cancer pathologies. Metabolomic profiling of EVs derived from prostate, cutaneous T-cell lymphoma, and colon cancer cell lines revealed elevated levels of proline and succinate compared to normal control samples. Additionally, EVs from prostate and cutaneous T-cell lymphoma cell lines exhibited increased concentrations of creatinine and folate. Adenosine was identified as a functional EV cargo in the BC cell line MDA-MB-231-luc-D3H2LN. Acting as an immunosuppressive molecule, adenosine binds to its receptors on CD8^+^ cytotoxic T lymphocytes, impairing their immunogenic activity and thereby facilitating immune evasion by cancer cells [121]. A recent study on EV metabolomics in prostate cancer explored the potential of EV-derived metabolites for early diagnosis and risk assessment. Key metabolites identified included L-glutamic acid, pyruvic acid, lactic acid, and methylmalonic acid, all implicated in cancer-specific metabolic reprogramming [122]. In addition to these findings, glyoxalase enzymes, particularly glyoxalase 1 (GLO1) and glyoxalase 2 (GLO2), have recently been identified as components of tumor-derived EV cargo, including those from breast cancer cells [123,124]. These enzymes are central regulators of methylglyoxal detoxification, a reactive byproduct of glycolysis. Their encapsulation in EVs, alongside other glycolytic enzymes, suggests a role in intercellular metabolic signaling and adaptation. Moreover, MG-H1 (methylglyoxal-derived hydroimidazolone), a major advanced glycation end product produced from methylglyoxal, has been found in the same EV populations. This metabolite may act as a paracrine signal that facilitates metastatic niche formation and colonization [125]. These findings highlight glyoxalases and their byproducts as potential novel mediators of cancer progression through EV-based communication. Clos-García et al. [126] investigated urinary EVs’ metabolomic profiles in patients with prostate cancer compared to those with benign prostatic hyperplasia, proposing a non-invasive strategy for monitoring disease progression. They identified 76 metabolites with significantly different concentrations between the two groups. Alterations involved acylcarnitines, kynurenine, citrate, and PC, along with a notable increase in 3β-hydroxyandrost-5-en-17-one-3-sulfate in prostate cancer patients. In BC, EVs have been shown to transport elevated levels of uridine and guanosine compared to those from non-tumorigenic cell lines [121]. Similarly, sEVs from head and neck squamous cell carcinoma samples contained increased levels of purine metabolites such as adenosine and inosine—both known for their immunosuppressive functions [127]. In ovarian cancer, EVs were found to be enriched in L-kynurenine, a metabolite that promotes angiogenesis by inducing endothelial mitophagy, thereby contributing to metastatic potential [128]. Although research on the metabolite cargo of EVs in cancer is relatively recent, interest in this area is rapidly growing. The expanding list of EV-associated metabolites with diagnostic and prognostic relevance highlights their emerging role in oncological disease management. Nevertheless, further studies are needed to standardize methodologies and validate clinical applications.

## 5. EVs Derived from BC Cells

The diagnosis of BC remains challenging and often distressing for patients due to the invasive nature of tissue biopsies, which are essential for accurate disease classification and therapy planning. Imaging techniques such as sonography and mammography, followed by radiological assessment, can be time-consuming and are sometimes limited by suboptimal precision and the potential for human error. Current non-invasive prediagnostic methods, including the detection of biochemical markers in blood (e.g., Cancer Antigen 15-3, Carcinoembryonic antigen, Tissue/Mammary Cancer Antigen, etc.), are increasingly viewed as outdated and often lack sufficient accuracy.

In response, the scientific community is actively seeking diagnostic strategies that are simpler, faster, painless, more accurate, and—most importantly—less invasive. Among the most promising avenues is the analysis of EVs, which offer significant potential not only for early diagnosis but also for prognostic assessment and therapeutic decision-making in BC [129].

### 5.1. TNBC

TNBC is an aggressive subtype of BC defined by the absence of three key receptors: ER, PR, and human HER2. Due to the lack of these specific therapeutic targets, chemotherapy remains the primary treatment option, underscoring the urgent need for novel and more effective therapeutic strategies [130]. TNBC is further subclassified based on the expression of characteristic gene signatures (basal-like 1, basal-like 2, immunomodulatory, mesenchymal, mesenchymal stem-like and luminal androgen receptor).

The basal-like subtype, which represents approximately 10–20% of all BC cases, is defined by the expression of genes such as KRT5, CDH3, ID4, FABP7, KRT17, TRIM29, and LAMC2. This subtype is frequently associated with TP53 mutations and poor clinical prognosis. Another distinct subtype is claudin-low TNBC, which accounts for 12–14% of BC cases. In addition to lacking ER, PR, and HER2 expressions, this subtype is characterized by the expression of markers such as CD44 and SNAI3. Like the basal-like subtype, claudin-low TNBC exhibits a high frequency of TP53 mutations and is similarly associated with adverse clinical outcomes [131].

#### 5.1.1. RNAs in TNBC EVs

**Figure 3:**
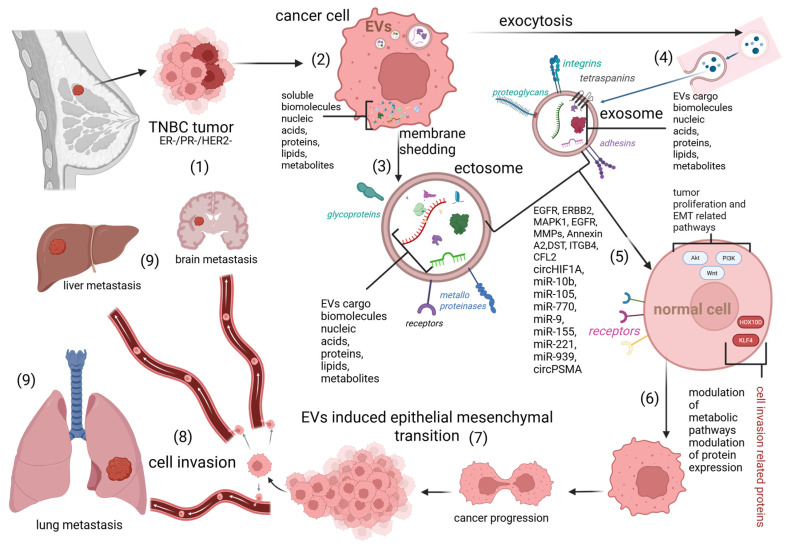
Simplified scheme of regulation and tumor progression via active regulatory biomolecules contained in EVs. (**1**) A breast lump represents a TNBC tumor, characterized by negative expression of PR, ER, and HER2+ receptors. (**2**) In the tumor cell, biogenesis of EVs occurs, involving the packaging of soluble regulatory factors directly into the cell membrane during membrane shedding (ectocytosis), resulting in ectosomes (microvesicles) that contain specific membrane molecules and cargo. (**3**) The biogenesis of EVs in cancer cells culminates in exocytosis, during which exosomes are secreted into the extracellular environment. (**4**) These exosomes are characterized by specific membrane molecules and contain cargo that includes regulatory molecules. Ectosomes, exosomes, and other EVs with cargo consisting of specific regulatory molecules such as miRNA, circRNA, and proteins are directed to their destinations, which may include normal cells. (**5**) Regulatory molecules target specific metabolic pathways and protein expression regulators in normal cells, thereby modulating metabolic pathways. (**6**) In this particular case, this modulation leads to cancer progression, EMT (**7**), and ultimately cell invasion (**8**), resulting in metastasis to distant organs (**9**).

EVs derived from TNBC cells contain bioactive molecules, including miRNAs, that contribute to tumor progression and metabolic reprogramming (Figure 3 and Table 2). One such molecule is miR-9-5p, which plays a significant role in CHL metabolism by targeting the genes INSIG1, INSIG2, and ATF3. In a BC mouse model, EV-associated miR-9-5p was found in significantly higher quantities compared to non-cancer controls, highlighting its potential as a cancer-specific metabolic regulator [132]. Another key molecule is miR-155-5p, a well-known oncomiR, which is upregulated in EVs released from BC cells. This miRNA is implicated in tumor initiation by activating the WNT signaling pathway through the downregulation of Adenomatous Polyposis Coli, a tumor suppressor gene. This suppression leads to pathway activation and promotes cancer progression. Additional targets of miR-155-5p include HSD17B12, MYC, SMAD1, SMAD3 and p53-inducible nuclear protein 1 (TP53INP1) all of which are downregulated, contributing to tumor advancement [133,134,135]. Furthermore, a study by Li et al. [136] demonstrated that miR-155-5p promotes both tumor progression and resistance to therapy by directly targeting TP53INP1, another tumor suppressor gene. In the study of Chen et al. [137], circHIF1A packed into EVs was identified, which plays a critical role in the growth and metastasis of TNBC. According to the study by Buschmann et al. [138], the overexpression of the glucocorticoid receptor significantly influences the miRNA composition of EVs derived from TNBC cells. In their analysis of EVs produced by the MDA-MB-231 and MDA-MB-468 TNBC cell lines, the most abundantly expressed miRNAs were identified as follows: MDA-MB-231: miR-100-5p, miR-21-5p, let-7f-5p, let-7i-5p, miR-486-5p, let-7a-5p, miR-92a-3p, let-7g-5p, miR-451a, and miR-27b-3p; MDA-MB-468: let-7f-2-3p, miR-103b, miR-4742-3p, let-7a-3p, miR-505-3p, let-7f-5p, let-7i-3p, miR-22-5p, let-7b-3p, and miR-196b-5p.

Additionally, miR-4516 was found to be significantly downregulated in CAF-derived EVs isolated from invasive ductal carcinoma samples, showing a fivefold decrease compared to normal tissue-derived EVs. This reduction suggests a potential tumor-suppressive role for miR-4516 in the TNBC microenvironment. Overexpression of miR-4516 in TNBC cells has been shown to inhibit cell proliferation. Its target gene, FOSL1, a transcription factor involved in tumor progression, was found to be overexpressed in TNBC cells, suggesting a tumor-suppressive role for miR-4516 [139]. A broader group of miRNAs associated with cancer progression and metastasis in TNBC-derived EVs includes miR-770, miR-9, miR-155, miR-221, miR-939, and the circRNA circPSMA1 [140]. In a study by Singh et al. [141], high levels of miR-10b were identified in EVs derived from TNBC cell lines. Upon uptake by recipient cells, miR-10b downregulated its target genes HOXD10 and KLF4, leading to enhanced cell invasion. Zhou et al. [142] further demonstrated that miR-105 is highly expressed in EVs derived from TNBC cells. This miRNA contributes to metastatic dissemination and vascular permeability in distant organs, promoting tumor progression. In a study by Ozawa et al. [143], the diagnostic potential of four EV-associated microRNAs—miR-142-5p, miR-150-5p, miR-320a, and miR-4433b-5p—was evaluated. These miRNAs were able to distinguish between Luminal A and TNBC subtypes, with miR-150-5p notably downregulated in EVs from TNBC samples. Furthermore, miR-421-5p was significantly overexpressed in TNBC-derived EVs and demonstrated high accuracy in differentiating TNBC from Luminal A cases, suggesting its value as a potential diagnostic biomarker. Another study investigated EV-derived miRNAs as prognostic indicators for recurrence in TNBC patients. miR-150-5p, miR-576-3p, and miR-4665-5p were found to be significantly upregulated in patients who experienced recurrence, highlighting their potential as non-invasive biomarkers for monitoring disease progression and guiding therapy decisions [144].

**Table 2:** TNBC EV-associated RNA bioactive molecules.

EV Cargo	TNBC	Function	References
RNA	miR-9-5p	CHL metabolism	[132]
RNA	miR-155-5p	Cancer initiation, WNT signaling pathway activation, tumor progression, drug resistance	[133,134,135,136]
RNA	miR-4516	Inhibition of cell proliferation	[139]
RNA	miR-10b	Promotion of cell invasion	[141]
RNA	miR-105	Metastatic and vascularization processes	[142]
RNA	miR-142-5p	Diagnostic potential for BC subtype differentiation	[143]
RNA	miR-150-5p	Diagnostic potential, downregulated in TNBC EVs compared to Luminal A	[143]
RNA	miR-576-3p	Prognostic marker for recurrence in TNBC	[144]
RNA	miR-4665-5p	Prognostic marker for recurrence in TNBC	[144]
RNA	miR-421-5p	Discrimination between TNBC and Luminal A patients	[143]
RNA	miR-100-5p, miR-21-5p, let-7f-5p, let-7i-5p, miR-486-5p, let-7a-5p, miR-92a-3p, let-7g-5p, miR-451a, miR-27b-3p	Significant expression in MDA-MB-231 cell line EVs, glucocorticoid receptor overexpression	[138]
RNA	let-7f-2-3p, miR-103b, miR-4742-3p, let-7a-3p, miR-505-3p, let-7f-5p, let-7i-3p, miR-22-5p, let-7b-3p, miR-196b-5p	Significant expression in MDA-MB-468 cell line EVs, glucocorticoid receptor overexpression	[138]
RNA	miR-770, miR-9, miR-155, miR-221, miR-939	Metastatic processes	[140]
RNA	circHIF1A	Growth and metastasis	[137]
RNA	circPSMA	Metastatic processes	[140]

#### 5.1.2. DNA in TNBC EVs

mtDNA is among the nucleic acid types identified in EVs derived from TNBC samples (Table 3). According to Vikramdeo et al. [145], EV-associated mtDNA in TNBC patients often harbors mutations, which may broaden current approaches to early diagnostics and preventive care. The study identified a total of 168 mtDNA mutations across all patient samples, with 73% located in coding regions and 27% in non-coding regions. Among these mutations, 80% were nucleotide transitions and 20% were transversions, with the G→A transition being the most frequently observed event. Each patient exhibited at least three distinct mutations. Mutations in non-coding regulatory regions, specifically RNR1 and RNR2, were detected in 15% of patients, while 12% of the mutations affected mitochondrial tRNA genes. Overall, mutation frequency in non-coding regions was low (around 3%), except for a few notable exceptions. For instance, the G1888A mutation was observed in 6% of patients. Among the tRNA-specific mutations, C5720T and T4434G were detected in 16% and 20% of patient samples, respectively. The remaining mutations were in coding regions encoding components of the mitochondrial respiratory chain:

- Complex I: MT-ND1, ND2, ND3, ND4, ND4L, ND5, ND6;
- Complex III: MT-CYTB;
- Complex IV: MT-CO1, CO2, CO3;
- Complex V: MT-ATP6, ATP8.

Mutations in these regions accounted for 44% of all mtDNA mutations. Notably, within Complex III, the T14894G mutation was found in 20% of the Complex III-specific mutations, far exceeding the average mutation rate of 3% in this region. In Complex IV, the T7953G mutation exhibited a remarkably high prevalence of 38%, compared to other mutations in the region, which ranged from 3% to 20% [145]. Another study demonstrated that EV-mediated transfer of mitochondria, specifically mutated mtND4, contributes to tumorigenesis and enhances chemoresistance in TNBC cells [146]. Furthermore, an intriguing study investigating the role of human papillomavirus (HPV) in TNBC found HPV DNA within EVs isolated from patient samples. These EVs could transfer viral DNA to stromal cells, which then exhibited an activated phenotype characterized by the expression of IL-6 and CD44. This transformation enhanced their ability to form mammospheres, suggesting a potential mechanistic link between HPV infection and TNBC initiation [147].

#### 5.1.3. Proteins in TNBC EVs

According to Mashouri et al. [148], EVs originating from TNBC cells transport tumor-promoting proteins, such as EGFR and matrix metalloproteinases (MMPs), to target cells, boosting their ability to invade and migrate. The study by Raiter et al. [149] discusses the Gal3 binding protein in isolated sEVs in TNBC samples where the Gal3BP/Gal3 complex secreted via EVs induces immunosuppression through the CD45 receptor. According to the study by Desai et al. [150], Annexin A2, a calcium (Ca^2+^)-dependent phospholipid-binding protein which is transported within EV cargo, is involved in TNBC metastasis. In a different study, the cargo of EVs derived from MDA-MB-231 cells was investigated, revealing the presence of EGFR, ERBB2, and MAPK1, which are involved in the PI3K/Akt signaling pathway that promotes metabolism, proliferation, cell survival, and growth. These compounds may all phosphorylate PKM2, leading to the activation of aerobic glycolysis, more aggressive cell proliferation, and activation of the EMT [151]. In the article by Risha et al. [152], three potential extracellular vesicle membrane/surface proteins were identified as markers for TNBC. Specifically, these proteins are glypican-1, glucose transporter-1, and disintegrin and metalloproteinase domain-containing protein 10 (ADAM10). Xu et al. [153] identified various proteins isolated from EV cargo derived from TNBC, which are related to various cell metabolism regulatory processes, specifically, platelet activation-related proteins TLN2, VASP, and GNAS; antigen processing and presentation-related proteins B2M and PSMB9; regulation of actin cytoskeleton-related proteins CFL2, ITGB4, and GIT1; angiogenesis-related proteins CEACAM1, COL4A2, and CALD1; and cell motility-related proteins DST, ITGB4, and CFL2147 (Table 4).

#### 5.1.4. Lipids in TNBC EVs

Lipids are a crucial component of cell signaling and, together with EVs, form a complex transport and signaling network in both physiological and pathological conditions within the organism (Table 5). Therefore, investigating their role in TNBC could be an essential factor in improving diagnostics, prognostics, and therapies for cancerous diseases. According to a study by Vikrameo et al. [145], the volume of cardiolipin associated with EVs from TNBC patient samples was significantly elevated. In another study, EVs derived from TNBC tumors were confirmed to traffic arachidonic acid, which plays a role in the reprogramming of neutrophils. This process leads to the accumulation of lipid droplets in cancer cells, thereby fostering an immunosuppressive tumor microenvironment [154]. In the study by D’Mello et al. [155], LysoPC 22:6/0:0 was proposed as an emerging biomarker for the diagnosis of TNBC.

#### 5.1.5. Metabolites in TNBC EVs

Two metabolites, one of which is mentioned in the lipid TNBC section, were proposed as innovative biomarkers in relation to the diagnosis of TNBC, specifically the metabolite N-acetyl-L-phenylalanine, which was found to be unique to the MDA-MB-231 cell line investigated in the study [155].

### 5.2. Luminal A

Luminal A BC accounts for 50–60% of BC subtype prevalence worldwide. This molecular subtype is characterized by the expression of ER, PR, and the lack of expression of HER2-. Moreover, it is also characterized by the low expression of Ki-67. It is characterized by the following genes: ESR1, GATA3, KRT8, KRT18, XBP1, FOXA1, TFF3, CCND1, and LIV1. This subtype has a low prevalence of TP53 mutations, and patients generally have a good prognosis for recovery [131].

#### 5.2.1. RNAs in Luminal A Subtype EVs

In the previously mentioned study by Ozawa et al. [143], it was found that miR-320a was overexpressed in Luminal A BC patient EVs compared to normal controls.

#### 5.2.2. Proteins in Luminal A Subtype EVs

According to the study by Xu et al. [153], proteins isolated from EVs derived from Luminal A BC were characterized through proteomic analysis. A specific set of proteins was found to be associated with this BC subtype. The identified proteins include those related to proteolysis, such as SDCBP, COLEC11, and LTF; proteins involved in protein folding, including CCT2, HSP90AA1, and PPIG; proteins associated with the regulation of necroptotic cell death, namely HSP90AA1 and SDCBP; proteins linked to cellular stress responses and proteins associated with cellular responses to external stimuli such as HSBP1, IL6, and HSP90AA1 (Table 6).

### 5.3. Luminal B

Luminal B BC is characterized by the variable expression and absence of specific receptors in various combinations (ER+/−, PR+/−, HER2−/+), along with the expression of particular genes (ESR1, GATA3, KRT8, KRT18, XBP1, FOXA1, TFF3, SQLE, LAPTM4B). High expression of Ki-67 was also recorded. This subtype exhibits an intermediate incidence of TP53 mutations and accounts for 10–20% of all BC cases. Based on gene expression profiles and other molecular characteristics, Luminal B BC is associated with an intermediate to poor prognosis [129].

#### Proteins in Luminal B Subtype EVs

According to the study by Xu et al. [153], the following set of proteins isolated from EVs derived from Luminal B BC was characterized through proteomic analysis: MYL6, AFDN, tight junction-associated proteins, extracellular matrix-related receptor (ECM–receptor) interaction-associated proteins RELN and GP1BB, fructose and mannose metabolism-related proteins SLC2A14 and SLC2A3, glucose metabolism-related proteins SLC2A14 and SLC2A3, and insulin signaling pathway-associated proteins PKLR and PYGM (Table 7).

### 5.4. HER-2+

HER-2+ BC is characterized by expression of the HER receptor and accounts for 10–15% BC incidence. This subtype is characterized by the following specific genes: ERBB2, GRB7, and ITGA7. The rate of mutations in the TP53 region is low and prognosis of the disease is intermediate [129].

#### 5.4.1. RNAs in HER-2+ Subtype EVs

According to the study by Chen et al. [9], several microRNAs are enriched in the EVs of the HER-2+ BC subtype. miR-29b-3p, miR-34c-5p, miR-203a-3p, miR-378g, and miR-382-5p are all involved in BC progression and, based on previous studies not focusing on EVs, are known as potential BC markers. However, this study had its limitations, as it utilized humanized mouse models, which may obscure the detection specificity for “only” human microRNAs. Nevertheless, the inclusion of next-generation sequencing and various non-coding RNAs, such as circular RNAs (circRNAs) and long non-coding RNAs (lncRNAs), may help address the speculated shortcomings of the method.

#### 5.4.2. Proteins in HER-2+ Subtype EVs

Xu et al. [153] isolated the following proteins from EVs from HER-2+ BC: cellular response to hydrogen peroxide-related proteins SRC, ARG1, MAPK13, and ErbB2/ErbB3; signaling event-related proteins DOCK7 and SRC; keratinization-related proteins CDSN, KRT78, and KRT23; tyrosine metabolism-related proteins FAH and ADHFE1; and arginine and proline metabolism-related proteins ARG1 and RARS1 (Table 8).

## 6. Conclusions

- EVs derived from BC cells have emerged as key players in tumor progression and metastasis, offering promising potential in modern diagnostics and therapeutic strategies. These vesicles, which carry diverse bioactive molecules such as RNA, DNA, proteins, lipids, and metabolites, are integral to intercellular communication and the modulation of the tumor microenvironment. EVs facilitate processes like immune evasion, angiogenesis, and metastatic niche formation, underscoring their significance in BC pathophysiology. Notably, EV cargo varies across BC subtypes, including TNBC, Luminal A, Luminal B, and HER-2+, reflecting unique molecular profiles that can be exploited for precision diagnostics and targeted therapies.
- Despite their transformative potential, EV-based liquid biopsy diagnostics for BC remain experimental and unstandardized. Current findings highlight the relevance of EV cargo in subtype-specific BC characterization, yet comprehensive references for all bioactive molecules in each subtype are limited. Advances in EV research could pave the way for non-invasive diagnostic tools, enabling early detection, subtype differentiation, and real-time monitoring of disease progression. Continued exploration of EVs and their molecular cargo holds promise for revolutionizing BC management, offering hope for improved patient outcomes through precision oncology.

Future research must focus on refining EV-based diagnostics to enable non-invasive, precise, and personalized cancer management. By leveraging the unique molecular cargo of EVs, innovative diagnostic tools can be developed to classify BC subtypes with greater accuracy, monitor disease progression, and predict therapeutic responses.

- As per the current understanding of BC and extracellular vesicle EV-based potential clinical applications, we recommend the following future directions for improved diagnostics, prognostics, and therapeutics:
  (1) Standardized EV isolation and profiling: Developing robust and reproducible protocols for EV isolation, such as advanced microfluidic systems and immunoaffinity techniques, will enhance the consistency and scalability of EV-based diagnostics. Improved characterization methods using next-generation sequencing, mass spectrometry, and metabolomics can uncover novel subtype-specific biomarkers.
  (2) Integration of artificial intelligence: Employing AI and machine learning algorithms to analyze EV molecular data could accelerate biomarker discovery, improve subtype classification, and predict patient outcomes with high precision. This approach will enable the identification of complex diagnostic patterns in EV cargo.
  (3) Multiplex panels for subtype differentiation: Comprehensive profiling of EV cargo across BC subtypes can facilitate the creation of multiplex panels for early detection, subtype classification, and therapy monitoring. These panels could combine RNA, DNA, protein, lipid, and metabolite biomarkers to improve diagnostic accuracy.
  (4) Therapeutic modulation of EV biogenesis: Investigating methods to modulate EV production and cargo content could lead to novel therapeutic strategies. For example, engineering EVs for targeted drug delivery or disrupting EV-mediated oncogenic signaling pathways could reduce tumor aggressiveness and metastasis.
  (5) Clinical validation and trials: Expanding large-scale clinical trials to validate EV-based biomarkers and diagnostic tools is critical for translating experimental findings into clinical practice. Collaborations between research institutions, industry, and healthcare providers can accelerate the development of EV-based technologies.
  (6) Subtype-specific therapeutic approaches: Tailoring therapies based on the molecular cargo of EVs in specific BC subtypes, such as TNBC or HER-2+, could improve treatment efficacy. For example, targeting EVs enriched with oncogenic RNAs or proteins in TNBC could offer new therapeutic avenues.
- By addressing these research and development priorities, EVs could become central to precision oncology, transforming the landscape of BC management and improving patient outcomes.

## Figures and Tables

**Table 1:** Overview of EV cargo in cancer.

EV Cargo Type	Examples	Mechanisms/Impact	Cancer Types
RNA	lncRNA H19, miR-181a-5p, circEHD2, miR-29a-3p	Regulation of metastasis, drug resistance, tumor-promoting inflammation	Lung, CRC, RCC, bladder
DNA	dsDNA (KRAS, TP53), mtDNA, c-Myc	Stable detection of mutations, tumor-specific DNA for liquid biopsy	Melanoma, pancreatic, colorectal
Proteins	CDCP1, CD9, TGF-β co-receptors, TYRP2, HSP70	Modulation of angiogenesis, metastasis, immune evasion	Prostate, melanoma, breast, HNSCC
Lipids	Phosphatidylserine, ceramide, glycosphingolipids	Biomarker potential, metabolic regulation, cancer-specific lipid signatures	Prostate, RCC, lung
Metabolites	Proline, adenosine, L-kynurenine, MG-H1, glyoxalase 1/2	Immunosuppression, metabolic reprogramming, angiogenesis	Prostate, breast, ovarian, colon, HNSCC

**Table 3:** TNBC EV-associated DNA bioactive molecules.

EV Cargo	TNBC	Function	References
DNA	mtDNA	Mutated mtDNA associated with early-stage cancer diagnostics and preventive care	[145]
DNA	mtDNA (RNR1, RNR2)	Regulatory region mutations detected in 15% of patient samples	[145]
DNA	mtDNA (tRNA genes)	Mutations specific to tRNA genes, impacting tumorigenesis and chemoresistance	[145]
DNA	mtDNA (G1888A mutation)	Mutation detected in 6% of patient samples	[145]
DNA	mtDNA (C5720T mutation)	Mutation present in 16% of patient samples	[145]
DNA	mtDNA (T4434G mutation)	Mutation present in 20% of patient samples	[145]
DNA	mtDNA (Respiratory Complex I)	Mutations in MT-ND1, ND2, ND3, ND4, ND4L, ND5, ND6 regions associated with mitochondrial respiratory chain	[145]
DNA	mtDNA (Respiratory Complex III)	Mutations in MT-CYTB, specifically T14894G mutation accounting for 20%	[145]
DNA	mtDNA (Respiratory Complex IV)	Mutations in MT-CO1, CO2, CO3 regions, with T7953G mutation exhibiting 38% prevalence	[145]
DNA	mtDNA (Respiratory Complex V)	Mutations in MT-ATP6 and ATP8 subunits	[145]
DNA	mtND4 gene	Increased mtDNA levels mutated in mtND4 gene transported in EVs responsible for tumorigenesis and chemoresistance	[146]
DNA	HPV DNA	Prevalence of HPV DNA in EVs linked to stromal cell activation and mammosphere formation	[147]

**Table 4:** TNBC EV-associated protein bioactive molecules.

EV Cargo	TNBC	Function	References
Proteins	EGFR, MMPs	Tumor-promoting proteins that enhance invasion and migration	[148]
Proteins	Gal3BP/Gal3 complex	Induces immunosuppression via the CD45 receptor	[149]
Proteins	Annexin A2	Involved in TNBC metastasis	[150]
Proteins	EGFR, ERBB2, MAPK1	Promotes PI3K/Akt signaling pathway, metabolism, proliferation, cell survival, EMT activation	[151]
Proteins	Glypican-1, glucose transporter-1, ADAM10	Potential EV membrane/surface markers for TNBC	[152]
Proteins	TLN2, VASP, GNAS	Platelet activation-related proteins	[153]
Proteins	B2M, PSMB9	Antigen processing and presentation-related proteins	[153]
Proteins	CFL2, ITGB4, GIT1	Regulation of actin cytoskeleton-related proteins	[153]
Proteins	CEACAM1, COL4A2, CALD1	Angiogenesis-related proteins	[153]
Proteins	DST, ITGB4, CFL2	Cell motility-related proteins	[153]

**Table 5:** TNBC EV-associated lipid bioactive molecules.

EV Cargo	TNBC	Function Specification	References
Lipids	Cardiolipin	Elevated levels associated with TNBC patient samples	[145]
Lipids	Arachidonic acid	Reprograms neutrophils, leading to lipid droplet accumulation and fostering an immunosuppressive tumor microenvironment	[155]
Lipids	LysoPC 22:6/0:0	Proposed as a new biomarker for TNBC diagnostics	[155]

**Table 6:** Luminal A-associated protein bioactive molecules.

EV Cargo	Luminal A	Function	References
Proteins	SDCBP, COLEC11, LTF	Proteolysis-related proteins	[153]
Proteins	CCT2, HSP90AA1, PPIG	Protein folding-related proteins	[153]
Proteins	HSP90AA1, SDCBP	Regulation of necroptotic cell death	[153]
Proteins	HSBP1, IL6, HSP90AA1	Cellular stress response and responses to external stimuli	[153]

**Table 7:** Luminal B-associated protein bioactive molecules.

EV Cargo	Luminal B	Function	References
Proteins	MYL6, AFDN	Tight junction-associated proteins	[153]
Proteins	RELN, GP1BB	ECM–receptor interaction-related proteins	[153]
Proteins	SLC2A14, SLC2A3	Fructose and mannose metabolism-related proteins	[153]
Proteins	SLC2A14, SLC2A3	Glucose metabolism-related proteins	[153]
Proteins	PKLR, PYGM	Insulin signaling pathway-associated proteins	[153]

**Table 8:** HER-2+-associated protein bioactive molecules.

EV Cargo	HER-2+	Function	References
Proteins	SRC, ARG1, MAPK13	Cellular response to hydrogen peroxide	[153]
Proteins	DOCK7, SRC	ErbB2/ErbB3 signaling event-related proteins	[153]
Proteins	CDSN, KRT78, KRT23	Keratinization-related proteins	[153]
Proteins	FAH, ADHFE1	Tyrosine metabolism-related proteins	[153]
Proteins	ARG1, RARS1	Arginine and proline metabolism-related proteins	[153]

## Data Availability

Not applicable.

## Abbreviations

The following abbreviations are used in this manuscript:

ADAM10	Metalloproteinase domain-containing protein 10
BC	Breast cancer
CAFs	Cancer-associated fibroblasts
CDCP1	CUB domain-containing protein 1
Cer	Ceramide
cfDNA	Freely circulating DNA
cf-mRNA	Cell-free mRNA
circRNA	Circular RNA
CRC	Colorectal cancer
dsDNA	Double-stranded DNA
ECM-receptor	Extracellular matrix-associated receptor
EGFR	Epidermal growth factor receptor
EMT	Epithelial–mesenchymal transition
ER	Estrogen receptor
EVs	Extracellular vesicles
HER2	Epidermal growth factor receptor 2
HPV	Human papillomavirus
CHL	Cholesterol
lncRNA	Long non-coding RNA
LysoPC	Lysophosphatidylcholine
MET	Mesenchymal–epithelial transition
miRNA	MicroRNA
MISEV	Minimal information for studies of extracellular vesicles
MMPs	Matrix metalloproteinases
mRNA	Messenger RNA
mtDNA	Mitochondrial DNA
MVEs	Multivesicular endosome
PC	Phosphatidylcholine
PR	Progesterone receptor
PS	Phosphatidylserine
RCC	Renal cell carcinoma
sEVs	Small extracellular vesicles
sncRNA	Small non-coding RNA
THSD7A	Thrombospondin type 1 domain-containing 7A
TNBC	Triple negative breast cancer
TP53INP1	p53-inducible nuclear protein 1
tRFs	tRNA-derived fragments
WHO	World Health Organization
